# Perceived Impacts, Acceptability, and Recommendations for Ecological Momentary Assessment Among Youth Experiencing Homelessness: Qualitative Study

**DOI:** 10.2196/21638

**Published:** 2021-04-06

**Authors:** Darlene Acorda, Michael Businelle, Diane Santa Maria

**Affiliations:** 1 Cizik School of Nursing The University of Texas Health Science Center at Houston Houston, TX United States; 2 Texas Children's Hospital Houston, TX United States; 3 Department of Family and Preventive Medicine University of Oklahoma Health Sciences Center Oklahoma City, OK United States; 4 Oklahoma Tobacco Research Center Stephenson Cancer Center Oklahoma City, OK United States

**Keywords:** youth experiencing homelessness, ecological momentary assessment, mobile apps, behavior change

## Abstract

**Background:**

The use of ecological momentary assessment (EMA) to study youth experiencing homelessness (YEH) behaviors is an emerging area of research. Despite high rates of participation and potential clinical utility, few studies have investigated the acceptability and recommendations for EMA from the YEH perspective.

**Objective:**

This study aimed to describe the perceived benefits, usability, acceptability, and barriers to the use of EMA from the homeless youth perspective.

**Methods:**

YEH were recruited from a larger EMA study. Semistructured exit interviews were performed using an interview guide that focused on the YEH experience with the EMA app, and included perceived barriers and recommendations for future studies. Data analyses used an inductive approach with thematic analysis to identify major themes and subthemes.

**Results:**

A total of 18 YEH aged 19-24 years participated in individual and group exit interviews. The EMA was highly acceptable to YEH and they found the app and EMA surveys easy to navigate. Perceived benefits included increased behavioral and emotional awareness with some YEH reporting a decrease in their high-risk behaviors as a result of participation. Another significant perceived benefit was the ability to use the phones for social support and make connections to family, friends, and potential employers. Barriers were primarily survey and technology related. Survey-related barriers included the redundancy of questions, the lack of customizable responses, and the timing of survey prompts. Technology-related barriers included the “freezing” of the app, battery charge, and connectivity issues. Recommendations for future studies included the need to provide real-time mental health support for symptomatic youth, to create individually customized questions, and to test the use of personalized motivational messages that respond to the EMA data in real time.

**Conclusions:**

YEH are highly receptive to the use of EMA in studies. Further studies are warranted to understand the impact of EMA on YEH behaviors. Incorporating the YEH perspective into the design and implementation of EMA studies may help minimize barriers, increase acceptability, and improve participation rates in this hard-to-reach, disconnected population.

## Introduction

Every year, an estimated 4.2 million youths and young adults in the United States experience some form of homelessness [1]. Black youth, unmarried parenting youth, and gay, lesbian, bisexual, transgender, and queer (LGBTQ) youth are at a higher risk for experiencing homelessness compared with other groups [1-3]. Youth experiencing homelessness (YEH) have disproportionately higher rates of negative health outcomes, including disease, injury, substance use, and trauma [4-6]. They are also at increased risk for victimization and incarceration [7,8]. Consequently, mortality rates among YEH are 10 times higher than those of housed youth in the general youth population, with suicide and drug overdose being the leading causes of death [9,10].

The transient and hidden nature of youth homelessness make it challenging to conduct research with this marginalized population [11]. Unlike stably housed youth, YEH experience barriers to accessing health and social services [12,13]. Even when available, YEH are reluctant to engage with helpful services over concerns for loss of confidentiality and perceived provider stigma, prejudice, and discrimination [14,15]. In-depth interviews from the Voices of Youth Count initiative revealed that the need for YEH to protect their identity and their history of stressed, often toxic relationships with parents and other adults make them less inclined to interact with unfamiliar resources and institutions [16]. For many YEH, weighing out the risks and benefits of engaging with others is paramount because, from their perspective “nothing is for free” [16].

In recent years, the widespread availability of smartphones and advances in mobile technology have made it feasible to conduct ecological momentary assessment (EMA) studies among hard-to-reach populations including YEH [17-19]. EMA collects real-time data as individuals go about their daily lives. As a result, some of the benefits of EMA include a reduction in recall and memory biases, an increase in ecological validity, and the ability to capture intrasubject variability among factors that contribute to behaviors [20-22]. EMA has been extensively used in adult addiction research [23-25], pediatric and adult mental health disorders [26,27], and nutrition and physical activity studies [28,29].

Research shows that EMA is acceptable among stably housed youth, yet the receptiveness of YEH to EMA is not well understood. Although there are similarities in the experience of EMA between the two groups, YEH face different challenges that require unique approaches [30]. To start, recommendations for recruitment of stably housed youth emphasize using schools as a starting point, a source that is not available for many YEH studies [31]. Clinical studies in stably housed youth also rely heavily on clinical institutions, such as primary care clinics, as touchpoints throughout the study, but YEH often face barriers to accessing these institutions [32,33]. Although stably housed youth have reported technology-related issues and recommendations similar to YEH, the lack of availability of outlets to charge a phone and disruptions in connectivity are significant barriers not commonly reported in stably housed youth studies [31,34]. Considering the higher incidence of victimization, trauma, and mental health issues among YEH, they may perceive EMA questions differently; thus, strategies that balance a thorough assessment within the context of potentially traumatic experiences need to be explored. The risks associated with mobile health technology in this vulnerable group is also not well understood.

The few EMA studies conducted with YEH demonstrated high response rates and acceptability [6,18,35]. Compared to previous cross-sectional methods used to understand the daily experiences of this highly mobile group, EMA allows for a more naturalistic approach to studying YEH behavior and their environmental circumstances [36]. Using longitudinal EMA data, Suchting et al [37] expanded on our understanding of shelter use among YEH by predicting patterns of sheltering behaviors. They revealed that stress related to not having a place to stay and experiencing discrimination to be among some of the strongest predictors for YEH not staying in shelters. Santa Maria et al [6] used EMA to capture patterns of YEH rates of drug use and to identify modifiable risk factors for future interventions. In another EMA study, sexual urge and drug use predicted day-level sexual activity, and YEH primarily engaged in condomless sex, increasing their risk for HIV and other sexually transmitted infections [38].

Few studies have explored the usability and acceptability of mobile phone assessments among YEH. Using focus group interviews, Jennings et al [34] found that YEH perceived mobile phone health interventions as beneficial if they addressed their health concerns, maintained their confidentiality, and allowed them access to calling and texting features. YEH were also receptive to EMA using SMS text messaging and had several suggestions for the question format, including the ability to explain answers instead of being limited to preset responses [35]. These findings provide valuable recommendations for YEH EMA studies but more studies are needed to further explore EMA use in YEH and no studies have investigated the perceived impact of EMA on YEH behavior [17,34,39]. We conducted this study to address these gaps and to describe the perceived benefits, usability, acceptability, and barriers to the use of EMA from the YEH perspective.

## Methods

### Parent Study

Participants in this study were recruited from a parent EMA study examining real-time factors, such as urge, stress, sexual behaviors, and substance use. Youths (N=71) from the parent study were between 18 and 24 years of age, English speaking, experiencing homelessness or unstable housing, and recruited from shelters and drop-in centers serving YEH in 1 city in the southern United States [6]. Youths participated in up to 21 days of EMA that prompted them to complete a brief assessment that asked about stress, affect, sexual activity, substance use, and sheltering arrangements 5 times a day. More information on the parent study can be found in the original publications [6,40].

### EMA Survey and Technology

Participants received prompts to complete EMA surveys through a Samsung Galaxy Light smartphone with an Android 4.2 operating system (Google). The surveys took fewer than 5 minutes to complete, and youths were cued visually and audibly for 30 seconds for each assessment. Daily assessments required participants to recall the past 24 hours, and random assessments occurred 4 times a day and focused on assessing real-time affect including feelings of anger, guilt, irritability, shame, and sadness [6]. Urge to use drugs or engage in sex or high risk behaviors including stealing were also assessed. Aside from the assessments, the phones could be used to send and receive phone calls and text messages and provided access to the internet and social media. At the end of the 21-day study period, participants met with the study team to return the study mobile phones and receive grocery store gift cards valued up to US $95 depending on the percentage of random and daily EMAs completed.

### Recruitment

**Figure 1:**
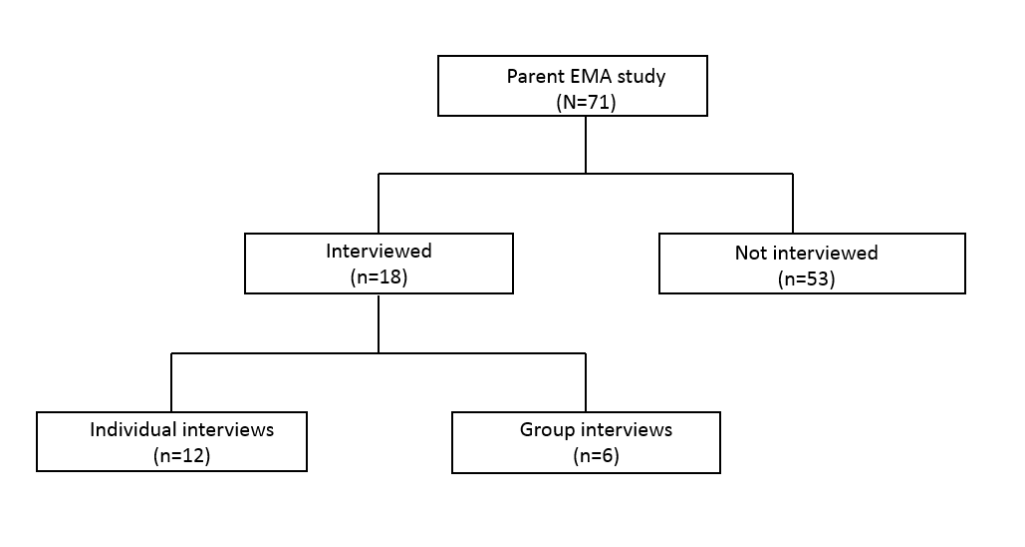
Recruitment. EMA: ecological momentary assessment.

After completion of the EMA study, youths were asked if they would like to participate in an exit interview individually or in a small group of peers. Interviews were conducted using a semistructured, conversational guide that inquired about their experience with the EMA app, what they liked and did not like about the app, and how they thought it impacted them. The interview guide was designed to address predetermined themes related to app acceptability and usability with open-ended questions to allow for emerging concepts. Finally, youths were asked if they would use the app again or recommend it to a friend and what they would change about the app or the study to make it better for others. Sessions were audio-recorded and transcribed verbatim. Ultimately, 14 interviews were conducted in total, including 2 group interviews (total of 6 participants) and 12 individual interviews (Figure 1). On average, both individual and group interviews lasted approximately 20 minutes.

### Analysis

An inductive approach guided the data analysis for this study. A team of 2 doctoral-trained investigators performed the analyses. First cycle coding included descriptive, attributive, in vivo, process and values coding [41]. Codes were then clustered into categories and patterns to generate an initial codebook. Second cycle coding focused on thematic analysis to identify themes and subthemes. The final codebook was refined and included definitions, exemplars, contrast quotes, and number of discussions identified for each theme. ATLAS.ti (version 7) was used to organize and code the data. Credibility and confirmability of the analysis was established through peer debriefing for each coding cycle, use of thick descriptions from the interview transcripts, and analytic memos during the analysis phase.

## Results

### Participant Characteristics

A total of 18 youths participated in this study (Table 1). Ages ranged from 19-24 years with a mean age of 21 years. For race/ethnicity, 11 out of 18 (66%) participants identified as male, 10 out of 18 (56%) identified as non-Hispanic Black or African American, 5 out of 18 identified as multiracial (28%), 1 identified as American Indian or Native Alaskan, and 1 participant reported “something else”. Most of the youths identified as heterosexual (14/18, 78%).

**Table 1:** Characteristics of youth experiencing homelessness (YEH) participants.

Youth	Age (years)	Gender	Sexual Orientation	Race
1	24	Male	Gay	Non-Hispanic Black or African American
2	21	Female	Heterosexual	Non-Hispanic Black or African American
3	19	Male	Heterosexual	Non-Hispanic White
4	22	Female	Heterosexual	Non-Hispanic Black or African American
5	20	Male	Heterosexual	Multiracial
6	20	Male	Heterosexual	Non-Hispanic Black or African American
7	24	Male	Gay	Non-Hispanic Black or African American
8	21	Male	Heterosexual	Multiracial
9	20	Female	Bisexual	Non-Hispanic Black or African American
10	21	Female	Heterosexual	Multiracial
11	20	Male	Heterosexual	Non-Hispanic Black or African American
12	24	Female	Heterosexual	Something else
13	19	Female	Lesbian	Multiracial
14	24	Male	Heterosexual	Multiracial
15	21	Male	Heterosexual	Non-Hispanic Black or African American
16	19	Male	Heterosexual	American Indian or Alaska Native
17	19	Female	Heterosexual	Non-Hispanic Black or African American
18	22	Male	Heterosexual	Non-Hispanic Black or African American

### Perceived Impacts of EMA

#### Increased Awareness of High Risk Behaviors

The majority of youths reported that the EMA increased their awareness of their high risk behaviors. The daily questions asking about their smoking habits, drug use, and sexual activity served as reminders of the frequency of their behaviors: “It kept me informed of how many drugs I use” (Y14). Another youth reported, “I would notice how many cigarettes I would smoke when I first started this survey, so that really made me like, okay, I really need to stop smoking those” (Y10). This youth further described how the repetition of questions led to increased behavioral and emotional awareness:

Yeah, it made me step back and look at everything because like I said, since it’s so redundant, it makes you notice your habits as well because you sit there, and you answer, and you start noticing I’m answering this question so many times this answer or this answer, and you start noticing. Then that makes you think to yourself, maybe I need to try and fix this because that’ll help me change my mood or whatever.

Y10

Four youths reported changing their behavior as a result of the EMA surveys. They described decreasing their substance use and taking precautions prior to engaging in any sexual activity: “It just make you think about certain things like you about to get ready to do something or it just makes you think or take precautions about what you’re doing” (Y7). One youth described decreasing his use of marijuana as a result of the survey:

I noticed that, you know, the marijuana really wasn’t helping. I still didn’t stop completely at that moment, but you know, down the line, I wean myself off of it. I’m still weaning myself off of it. I haven’t really smoked in like 2 weeks. So that’s good.

Y17

Youths reported making changes in their sexual behaviors with some ensuring they had a condom available at all times:

With the sex, it kind of helped me slow down a little. We were having sex—me and my girlfriend—and it did kind of slow us down a little bit because I noticed we were just having sex recklessly.

Y10

#### Increased Awareness of Emotional State

Six youths conveyed that the EMA surveys helped them be aware of their emotional state. Youths reported that the words in the questions allowed them to recognize and name their emotions:

It was like the way that they ask the questions, it helped me kind of put it in words because I would sometimes feel like fidgety or irritated. I’m like, I’m not really sure why. But then the way they would ask I was like “Ok, that’s probably why.” Maybe that’s the word I’m supposed to use.

Y3

Another youth described the survey as a way of coping with their emotions:

It help me be more aware of how I was feeling, because it was like you asked the right questions at the right time. So, it’s more like self-coping. Therapy in a way. Like, basically you’re being able to acknowledge how you are feeling, like what is causing that emotion that’s holding you down.

Y8

Youths described the benefits of acknowledging their stress and identifying emotional triggers: “It brings to your own attention that okay, well, a lot of the stress that I’m having is sometimes due to me or it’s sometimes due to things around me” (Y15). Another youth reported, “Most of my stress came from not just everyday life but mostly from how I put myself through life” (Y10). One youth described the impact of the survey on her stress and coping skills:

I mean, when it’s brought to your attention every day or when you basically put it in your own face every day because that’s what the survey’s doing. It’s basically letting you note to yourself. It brings it to your own attention that a lot of the stress I’m having is sometimes due to me or sometimes the things around me, so it helps you take a step back and see how you can attack each situation instead of just running into it blind.

Y10

#### Provided Social Support and Connection

For many youths, participating in the survey made them feel heard and supported:

Emotionally, I felt like there was actually somebody who cared or wanted to know how I felt. I like that.

Y18

They reported that the ability to not only express themselves but also the knowledge that someone was reading their responses was comforting. One youth described how the survey helped her cope with loneliness:

…it was kind of helpful to me because sometimes I had no one to talk to and then I be feeling like I can’t express, but when I take the survey and it ask like how you feeling or are you depressed. It make it seem like someone actually going to look at this and say, well, she’s feeling this type of way. Somebody actually sit down and cares about your feelings. That’s what I liked about the survey.

Y17

Seven youths explained that the ability to connect with friends and family through phone calls, text, and social media was most beneficial. One youth used the phone to reconnect with her family whom she had not spoken to for some time: “…haven’t talked to my mom in a long time. I finally gave her a call” (Y1).

### Acceptability of EMA

#### Provided a Distraction From Homelessness

In general, YEH were receptive to the EMA surveys. Some reported that the surveys distracted them from homelessness:

I be like trying to get my mind off things, like focus on something else. I’ll be like “Okay, well I’ll take my mind off this and focus on the surveys.”

Y18

Several reported it helped them avoid risky behaviors:

If I’m bored, I get adventurous. If I’m on the phone, I just be still stuck on the phone just watching movies or videos, anything, for hours.

Y12

YEH also expressed appreciation for being able to use the phones for other purposes including phone calls and social media:

You didn’t just give them a phone and oh, here are those surveys. Even though we are young kids, we have access to Facebook, we have access to things we actually do. They can entertain themselves, and they’re on the streets, so they can charge the phone and be on the phone all night, something for them to do. It’s not just doing surveys on this phone and still sitting outside bored.

Y15

#### Perception of Being Watched

Youths described instances in which they felt that the prompts came as a response to their real-time behavior. They mentioned feeling “watched over” or being monitored especially when the survey questions applied to their real-life situation: “Sometimes I felt like it was the signs; it was kind of weird because it would pop up only when I’m about to do something” (Y17). One youth described his experience after a police encounter:

One time I actually thought the phone knew what I was doing because 2 minutes later, I had got into it with the lawman and right after I finished talking to the lawman, the survey came up and its first question was “Did you encounter a lawman or a police officer?” I was like, “really, bro?”

Y12

#### Barriers to Acceptability and Engagement

Redundancy of questions was a frequently mentioned issue. As a result, youths described being able to predict the questions and answering without much thought to their responses: “Because I would get bored because it’s just like now, I know exactly what it’s going to say, and it’s like click, click, click” (Y13). One pregnant youth described that the lack of customized questions was a problem: “Only one time did I get something that asked if I was pregnant, and I was like yeah, and then it asked me the same exact questions it would ask me even if I wasn’t pregnant” (Y15). Some youths reported that the lack of response to their answers was dissatisfying. One youth described the following:

Let’s say, by the third time, I say I’m extremely stressed, maybe that could send y’all a red alert like, “Hey this person’s stressed. They might need someone to talk to.” Then you could send them a message like, “Are you okay? Want someone to talk to? Need anything?” I feel that would make certain individuals feel a little bit more looked after, a little more cared for. And it’s not just like, oh, it’s a survey…if you really need somebody, this is for us to find out.

Y13

### Usability of EMA

#### Easy to Use but With Glitches

Youths reported that the surveys were easy to navigate but that the questions were often predictable: “…it got to the point where my boyfriend, he knew every question, so he could just type” (Y15). Most youths found the redundancy of questions to be boring and not applicable to their specific situations. Although they did not mind the number of surveys administered daily, some of the youths found the timing of the surveys to be challenging: “If you’re not on it [phone] or not around it, it’ll go off and you hear it…and then you missed it” (Y2). A few of the youths reported “glitches” occurring while taking the surveys describing that the surveys would turn off midway leaving some questions unanswered: “…like it would pop up—sometimes a survey wouldn’t pop up; it would just go off, so that’s why some of them weren’t answered” (Y15).

#### Connection and Other Technology-Related Issues

Technology-related issues were common. Some described not being able to make phone calls: “…when I try to call out, it’ll say only numbers that can be dialed or numbers that are programmed” (Y12). Others reported issues with internet access: “When I first got the phone, it was going really fast; and then like two days after that, everything started slowing down…it got really, really slow” (Y11). Some stated that the slow internet access also slowed down their ability to send the completed surveys. Battery issues were commonly reported by youths who often struggle to find a place to charge their phones: “The app was good, just the phone was bull crap because it only worked for like 2 days, and then after that it just went down” (Y12).

#### YEH Recommendations for Future EMA Studies

The majority of youths recommended that the survey questions should be customized to each participant to avoid redundancy: “I don’t think each survey should say the same thing; I think you should switch it up” (Y15). YEH also expressed that although the questions about mental states and substance use were important, they would also like to be asked everyday life questions such as “How are you feeling today?” or “Are you hungry?” or “Did you work out today?” They also reported that there needed to be more questions and that questions should “go more deep into it [topics]” (Y9). Two youths expressed that they would have liked questions about their plans for the future and where they saw themselves in 5 years. Some of the youths recommended that the ability to type in their responses instead of choosing from preset answers would be preferable:

Yeah, type in your answer. “I feel upset because,”… I just feel like they shouldn’t be “sometimes,” “often,” “agree,” “disagree”. Have distinct answer.

Y15

Youths expressed that additional supports should be provided through the EMA surveys. However, they recommended that support should be personalized so that the participants feel “more cared for”. Describing this type of support, one youth stated the following:

Don’t read them a script. Literally sit there and ask them, why do you feel like this? Don’t suggest that they go to a mental hospital. Give them encouraging words, and talk to them for a minute, ask them do they feel safe at this moment, and if they don’t feel safe, then “Can you call the police for me?” “Can you call the ambulance?” And then take that step.

Y15

Three youths recommended that allowing customization of the phone ring tones may help with survey compliance. Others stated that allowing participants to go back and change their answers and to retroactively complete missed surveys would be helpful since surveys may come when they are too busy to respond, or they respond incorrectly:

I also think you should make it to where you can be able to go back and redo your answer. I think that is a big part of it because I just—I really like it. I really liked this. It’s a good thing to me.

Y15

To prevent phones from being stolen, several youths recommended that participants should be screened carefully prior to receiving their phones: “You just have to be pretty strict on who gets them…because people see that it’s just a regular phone and not something you have to do a duty on” (Y10). Many of the youths expressed that allowing the youth to keep the phone after the study would help with keeping them connected to social media and provide them access to resources.

Some youths found the timing of the surveys challenging and suggested set times for the survey to increase compliance. One youth indicated the following:

…what would be a little bit better is if the times were consistent because the one that I missed, I was on the internet looking up the bus route for something, and it came up, and I’m like in the middle of trying to find the bus route, and then my dad started calling, and then my phone just froze. I was like, if I knew it come at 10:30 and 12:15, I would know, okay, have my phone charged. And I think—and that’s why my boyfriend missed so many because he never knew when they were coming, but he never brought his charger. He was like, “Man, if I knew what time it was coming, I’d at least make sure my phone was charged for that time…”

Y13

## Discussion

### Principal Findings

The findings in this study suggest that EMA is acceptable to YEH and may have perceived behavioral benefits. Increased self-awareness as a result of receiving daily questions about their behaviors and emotions led some youths to decrease their substance use and increase condom use before sexual intercourse. Others expressed that the questions helped them recognize stressors that led to negative emotions, and as a result, youths intentionally avoided these emotional triggers in the future. Researchers have previously noted the impacts of frequent assessments on behavior and emotional awareness irrespective of interventions [42,43]. This effect, known as assessment reactivity, results from participants' constant reflection during repetitive behavior evaluations, leading to change [44]. EMA questions may serve as cues to action by providing information and reminders that trigger health-promoting behaviors [42]. However, previous EMA reactivity studies in alcohol abuse, smoking cessation, and medication adherence have found mixed results [44-47]. A randomized control trial evaluating EMA reactivity in smoking cessation discovered that although increased assessments did not promote abstinence, researchers observed small reactivity effects on anxiety, hunger, positive affect, and post-quit confidence [47]. To date, no studies have explored this concept in YEH, and it is unclear whether youths sustained their behavior change postintervention. Researchers should consider the effects of assessment reactivity in the design of future EMA studies to anticipate potential confounders in youths' subjective experiences and to assess for weaning effects over time.

Youths felt high levels of perceived support through their interactions with the EMA surveys. Although the surveys were pregenerated, administered at set times during the day, and unidirectional in nature, being asked about their day made youths feel heard and looked after. Tyler and Schmitz [48] observed similar findings in their SMS study with YEH, reporting “perceptions of care and concern” as one of the perceived benefits of participation. These discoveries highlight the degree of loneliness many YEH experience; interactions, even in survey form, appear to alleviate feelings of isolation and depression common in YEH [49]. In this regard, EMA may have unintended therapeutic effects on YEH’s mental well-being, particularly those with low social supports.

Youths unanimously reported that the most beneficial aspect of the EMA study was the ability to connect with friends, family, and other social support through the mobile phone. Access to the internet allowed youths to communicate with their peers through social media and to access multimedia platforms that helped alleviate boredom. Some of the youths used the phones for directions to clinics and grocery stores; one youth reported a friend getting a job interview through the phone. Many of the youths recommended that participants be allowed to keep the phones after the study. This is not surprising, as previous studies observed that mobile phones serve as lifelines for youth [11,39]. Aside from communicating with peers, they used phones to access resources and other forms of support [34]. However, mobile access can have adverse effects with youths expressing concerns for loss of anonymity and misuse of their personal information [34,39]. In this study, YEH did not report these concerns; however, several described feeling watched over and believed that the surveys occurred as a response to their real-time behavior and situation. Future EMA studies should consider the potential for harmful effects of mobile connectivity on YEH privacy; clearly outlining the plans for data management and measures to ensure confidentiality may help to alleviate these fears.

EMA was highly acceptable to YEH. Youths reported that the questions were easy to understand and that the frequency and length of the surveys were manageable. Some of the youths recommended that questions should “go deeper” and that participants should be allowed to provide contextual answers instead of preset responses. Youths also recommended that surveys should be customized to each individual and that redundancy of questions should be avoided. Several youths reported that redundancy led them to answer mindlessly after taking the same survey multiple times. These findings are similar to recommendations from other studies with YEH reporting that the authenticity of questions and the ability to explain their answers were most important [17,34]. YEH emphasized that authenticity may only be realized when they are directly involved in content development [34]. A collaborative approach to EMA study designs may improve participation and provide valuable insights into the YEH experience.

Although YEH reported that the surveys were easy to navigate, they encountered issues with connectivity, phone battery life, and timing. The stalling of the EMA app resulted in incomplete or missed surveys. Youths reported issues with the phone battery life despite frequent charging and experienced slower connectivity as the study progressed. Youths also described instances when they did not receive notification or were unable to complete the surveys on time. Other EMA studies have reported similar issues. Mackesy-Amiti and Booram [50] observed that notification issues (25%), stalling of the EMA app (17%), and battery issues (5%) accounted for most of the problems encountered in their study. A systematic review of the use of EMA methods with youth found that software and hardware malfunctions and connectivity issues were common across many EMA studies [51]. As technical issues can significantly impact data collection and ongoing participation, efforts should be made to provide ongoing technical support to help troubleshoot software and hardware problems. Piloting the phone and app prior to the start of the study may help mitigate potential issues; however, lost phones were a significant problem in this study, and piloting may not always be feasible in this population.

YEH had several recommendations for future EMA research. They emphasized the importance of providing real-time support for themselves and their peers during hard times. For example, youths identified during the EMA as having suicidal thoughts or who are feeling severe depressive symptoms could be connected to crisis personnel for help. Aside from additional support, youths also wished for “normal” interactions and more goal-related questions. For instance, they wanted to be asked how they were doing and about their plans for the future. These findings reflect the perceived stigma YEH experience; being seen as difficult or different from other "normal" youth are negative stereotypes [52]. Including questions unrelated to mental health or high risk behaviors may help to minimize this stigma and signal to YEH that they are valued as a whole individual. Youths also expressed the desire to receive motivational messages to help them through difficult times. Previous studies observed that youths were particularly interested in receiving motivational messages, tips on staying calm, and advice on how to navigate relationships [17,39]. Integrating these preferences into the EMA may improve acceptability and survey compliance.

This study aligns with current literature that EMA has high acceptability and utility with YEH. Several of the findings are similar to those found in stably housed youth; however, the unique challenges faced by YEH and the potential of EMA to impact high risk behaviors are highlighted in this study. Although findings suggest a positive perceived behavior impact, more studies are needed to examine the direct relationship between EMA and behavior change, the impact of assessment reactivity on EMA data, and the sustainability of change postintervention. This study adds to the literature by providing recommendations for EMA research from the perspectives of YEH. Future research is needed to identify strategies for involving YEH in EMA development and understanding barriers to participation and compliance. Future EMA studies would benefit from incorporating just-in-time adaptive messages that respond in real-time to EMA data and generate youth-developed motivational, health-promoting messages. Additional trials should be conducted to assess the behavioral impacts of EMA.

### Limitations

This study has several limitations. As this is a single-site study with a relatively small sample size, the generalizability of findings to other populations of YEH is limited. The experiences reported here may be unique to those who chose to participate in exit interviews; these youths may have had more positive or negative experiences, which may not be reflective of the experience of all the youths who participated in the original EMA study. There is also an underrepresentation of LGBTQ youth in the sample; estimates suggest that up to half of the general YEH population identify as LGBTQ [53]. As data were collected from both individual and group interviews, more vocal participants might have disproportionately impacted specific themes; however, all efforts to obtain exemplars from a diverse group of youth experiences were made by the researchers during the analysis and the reporting phases of the study.

### Conclusions

Findings from this study describe a positive perceived behavior impact on youth high risk behaviors; however, more studies are needed to fully understand the direct effect of EMA on behavior change among a larger and more diverse YEH population. EMA methods are highly acceptable to YEH, but technology and connectivity issues serve as significant barriers to participation and survey compliance. The development of future EMA studies should involve YEH to ensure the authenticity of surveys and address their specific needs.

## Abbreviations

EMA: ecological momentary assessment
LGBTQ: gay, lesbian, bisexual, transgender, and queer
YEH: youth experiencing homelessness
